# Spatial analysis of G.f.fuscipes abundance in Uganda using Poisson and Zero-Inflated Poisson regression models

**DOI:** 10.1371/journal.pntd.0009820

**Published:** 2021-12-06

**Authors:** Albert Mugenyi, Dennis Muhanguzi, Guy Hendrickx, Gaëlle Nicolas, Charles Waiswa, Steve Torr, Susan Christina Welburn, Peter M. Atkinson

**Affiliations:** 1 Coordinating Office for Control of Trypanosomiasis in Uganda, Ministry of Agriculture, Animal Industry and Fisheries, Kampala, Uganda; 2 School of Biomedical Sciences, Edinburgh Medical School, College of Medicine and Veterinary Medicine, The University of Edinburgh, Edinburgh, United Kingdom; 3 College of Veterinary Medicine Animal Resources and Biosecurity, Makerere University, Kampala, Uganda; 4 Avia-GIS, Zoersel, Belgium; 5 Department of Vector Biology, Liverpool School of Tropical Medicine, Liverpool, United Kingdom; 6 International Campus, ZJU-UoE Institute, Zhejiang University School of Medicine, Zhejiang University, Zhejiang, China; 7 Faculty of Science and Technology, Lancaster University, Lancaster, United Kingdom; Universidad de Buenos Aires, ARGENTINA

## Abstract

**Background:**

Tsetse flies are the major vectors of human trypanosomiasis of the form Trypanosoma brucei rhodesiense and T.b.gambiense. They are widely spread across the sub-Saharan Africa and rendering a lot of challenges to both human and animal health. This stresses effective agricultural production and productivity in Africa. Delimiting the extent and magnitude of tsetse coverage has been a challenge over decades due to limited resources and unsatisfactory technology. In a bid to overcome these limitations, this study attempted to explore modelling skills that can be applied to spatially estimate tsetse abundance in the country using limited tsetse data and a set of remote-sensed environmental variables.

**Methodology:**

Entomological data for the period 2008–2018 as used in the model were obtained from various sources and systematically assembled using a structured protocol. Data harmonisation for the purposes of responsiveness and matching was carried out. The key tool for tsetse trapping was itemized as pyramidal trap in many instances and biconical trap in others. Based on the spatially explicit assembled data, we ran two regression models; standard Poisson and Zero-Inflated Poisson (ZIP), to explore the associations between tsetse abundance in Uganda and several environmental and climatic covariates. The covariate data were constituted largely by satellite sensor data in form of meteorological and vegetation surrogates in association with elevation and land cover data. We finally used the Zero-Inflated Poisson (ZIP) regression model to predict tsetse abundance due to its superiority over the standard Poisson after model fitting and testing using the Vuong Non-Nested statistic.

**Results:**

A total of 1,187 tsetse sampling points were identified and considered as representative for the country. The model results indicated the significance and level of responsiveness of each covariate in influencing tsetse abundance across the study area. Woodland vegetation, elevation, temperature, rainfall, and dry season normalised difference vegetation index (NDVI) were important in determining tsetse abundance and spatial distribution at varied scales. The resultant prediction map shows scaled tsetse abundance with estimated fitted numbers ranging from 0 to 59 flies per trap per day (FTD). Tsetse abundance was found to be largest at low elevations, in areas of high vegetative activity, in game parks, forests and shrubs during the dry season. There was very limited responsiveness of selected predictors to tsetse abundance during the wet season, matching the known fact that tsetse disperse most significantly during wet season.

**Conclusions:**

A methodology was advanced to enable compilation of entomological data for 10 years, which supported the generation of tsetse abundance maps for Uganda through modelling. Our findings indicate the spatial distribution of the G. f. fuscipes as; low 0–5 FTD (48%), medium 5.1–35 FTD (18%) and high 35.1–60 FTD (34%) grounded on seasonality. This approach, amidst entomological data shortages due to limited resources and absence of expertise, can be adopted to enable mapping of the vector to provide better decision support towards designing and implementing targeted tsetse and tsetse-transmitted African trypanosomiasis control strategies.

## Author summary

Tsetse flies are the major vectors of human and animal trypanosomiasis. To support control efforts, there is need to understand the location of these vectors in terms of distribution and abundance. Due to limited resources and unsatisfactory technology, delimiting the extent and magnitude of tsetse coverage remains a challenge. In a bid to overcome these limitations, this study attempted to explore modelling skills that can be applied to inform decision makers about the status of tsetse abundance using limited historical tsetse data and a set of remote-sensed environmental variables. Two regression models; standard Poisson and Zero-Inflated Poisson (ZIP) were fitted and evaluated for superiority. The results indicated the level of responsiveness of each covariate in influencing the vector across the study area. Tsetse abundance was found to be largest at low elevations in areas of high vegetative activity, in game parks, forests and permanently flooded shrubs during the dry season. This approach can be adopted to enable mapping of any tsetse species to provide better decision support towards designing and implementing targeted tsetse and tsetse-transmitted African trypanosomiasis control strategies amidst entomological data shortages due to limited resources and absence of expertise.

## Introduction

The World Health Organization aims for the elimination of Human African Trypanosomiasis (HAT) as a public health problem by 2020 and for full elimination by 2030 [1–5]. One of the strategies to achieve this is by reducing the tsetse-human interaction [6]. Trypanosomiasis is a vector-borne disease of humans and animals caused by species of Trypanosoma transmitted by tsetse flies [7]. This implies that African trypanosomiasis (AT) is maintained by the interaction amongst three elements: vertebrate host (humans or livestock)[, Trypanosomes species and Glossina species [8,9]. Therefore, mapping the distribution and abundance of tsetse flies assists in improving the understanding of Trypanosomiasis risk and developing rational tsetse and AT control decisions.

Vector control programmes often make use of vector abundance and spatial distribution data sets to plan control interventions. Vector abundance, which is a measure of the vector population in a unit area and over a given time-scale, is commonly determined using detailed field survey data. Such data, especially for large areas, are often not readily available. In many cases, vector control programme managers depend on estimates of vector abundance obtained through application of probability-based sampling techniques using fine-resolution field vector survey count data from small areas to plan their activities [10,11]. Fine resolution data are key to ensuring delivery of reliable estimates of vector abundance within an area [12,13]. However, a major problem that has to be addressed explicitly when estimating vector abundance is false negatives: zero tsetse catches in areas where tsetse do exist [8]. Vectors do not always occupy all areas that are suitable for them, or are not always found even when they do occur, due to low abundance and chance [14]. Spatial heterogeneity in abundance within “suitable” areas impacts on disease transmission.

The abundance of a target vector species is a fundamental ecological parameter and a critical consideration when making vector management and control decisions. Location-specific tsetse count data collected by entomologists during tsetse control and monitoring programmes are good estimates of approximate levels of tsetse abundance in a given area. Sileshi [15] asserts that; “there are many kinds and levels of decisions that need to be made based on vector abundance”. For example, monitoring the performance of tsetse (*Glossina spp*.) control programmes largely depends on periodic measurements of tsetse abundance; tsetse abundance often determining appropriate tsetse control interventions.

*Glossina spp*. abundance; how common or rare a particular tsetse species or sub-species is in a defined location or community is a component of biodiversity and can be viewed from an ecological perspective. Vector abundance is regulated by abiotic factors (such as temperature, moisture of breeding habitat and humidity) and biotic factors (parasites, predators, vegetation and pathogens) and their interaction. These factors uniquely determine the spatial patterns of vector abundance [16]. Among abiotic factors, temperature and humidity are the most important factors that constrain Glossina spp. abundance and distribution [17]. Temperature regulates the ecology of Glossina spp. communities. Overall, *Glossina spp*. are very sensitive to climatic changes [18]. *Glossina spp*. (*G*. *pallidipes* and *G*.*m*.*morsitans*) abundance, for example, has been positively correlated with temperature [19]. Tsetse fly physiology and behaviour influence their abundance and spatial distribution. This study focused on the interaction of micro-climate and environment as represented by remote sensed surrogates in influencing tsetse abundance (*Glossina fuscipes fuscipes*). Taylor [20] contended that the landscape yields characteristic parameters that segregate species, and that these parameters are the population expression of the individual behaviour defined by the ethologists and observed by the naturalists. Like other organisms, *Glossina spp*. are linked to unique spatial patterns that are influenced by ecological settings. The environment influences the behaviour and physiology of *Glossina spp*., which then determines their spatial patterns.

*Glossina spp*. abundance maps have been constructed in some countries at very small scales; commonly 1:1,000,000. For example, during the period 1979–1980, Cote d’Ivoire with the support of the Food and Agriculture Organization of the United Nations (FAO) and German Technical Cooperation Agency (GTZ) collected Glossina spp. from sampled points and produced sub-national *Glossina spp*. abundance maps [14]. These have been a resource to the entomology sector in the country. Similar studies leading to mapping of *Glossina spp*. distribution and abundance have been conducted in East Africa [21], Togo [14], Zimbabwe [14], Kenya / Tanzania [14], Ghana [11] and Kenya [22].There are no records signifying any previous mapping leading to the production of National level Glossina spp. abundance maps in Uganda. At sub-national level efforts have been made to produce abundance maps for northern Uganda [23]. The current national *Glossina spp*. data available for Uganda [8,24] are explicitly on presence / absence predictions rather than abundanc.

## Methods

### Study area

Uganda is located along the Equator, positioned nearly at the centre of Africa, and is land-locked by DRC, Kenya, Rwanda, South Sudan and Tanzania. Although much of Uganda is a plateau, its altitude varies between 615 m to 5111 m above sea level. The country has a wide variety of tropical vegetation, abundant seasonal rainfall and plenty of water stored in lakes, rivers and swamps. Over 70% of the country is estimated to be tsetse infested [8]. Due to the widespread presence of tsetse flies, over 11 million people and 10million cattle in the country are at risk of contracting trypanosomiasis.

The livestock sector contributes 17% of the agricultural gross domestic product. As such, 3.8 million households (58% of the entire 40 million national population) draw their direct livelihoods from the livestock sector [23]. The majority of the population lives in rural areas and, thus, most people earn their living through direct interaction with the natural environment (farming, fishing, forestry, mining, hunting etcetera). It should be observed that while close to 80% of the country is arable, the majority of farming is subsistence agriculture, highly dependent on natural rainfall and use of traditional farming technologies. The burden of tsetse-transmitted trypanosomiasis affects the livelihoods of many rural communities in the country both directly and indirectly [25,26].

Historically, most tsetse fly control operations were initiated and implemented by the central government. Key operations included; bush clearing, game elimination through hunting, game eviction, cattle evacuation, maintenance of tsetse pickets, application of insecticide on cattle, and ground spraying using Dieldrin-3% [27,28]. These interventions were geared towards removal of the tsetse habitat or direct killing of the tsetse flies themselves. While these interventions had a significant impact on creation of tsetse free zones at the time, the approaches, especially those involving bush clearance and game elimination, caused environmental degradation [27,29–33]. Hence, they have since been discontinued due to environmental concerns. Recently, most successful tsetse interventions have been carried out under ‘project conditions with substantial funding. Unfortunately, sustaining achievements in most cases has been a great challenge since projects are usually short-lived [18]. The response has been to promote community-based interventions involving animal spraying by farmers themselves and tsetse trap deployment by community members [34,35]

### Tsetse fly count data

**Fig 1:**
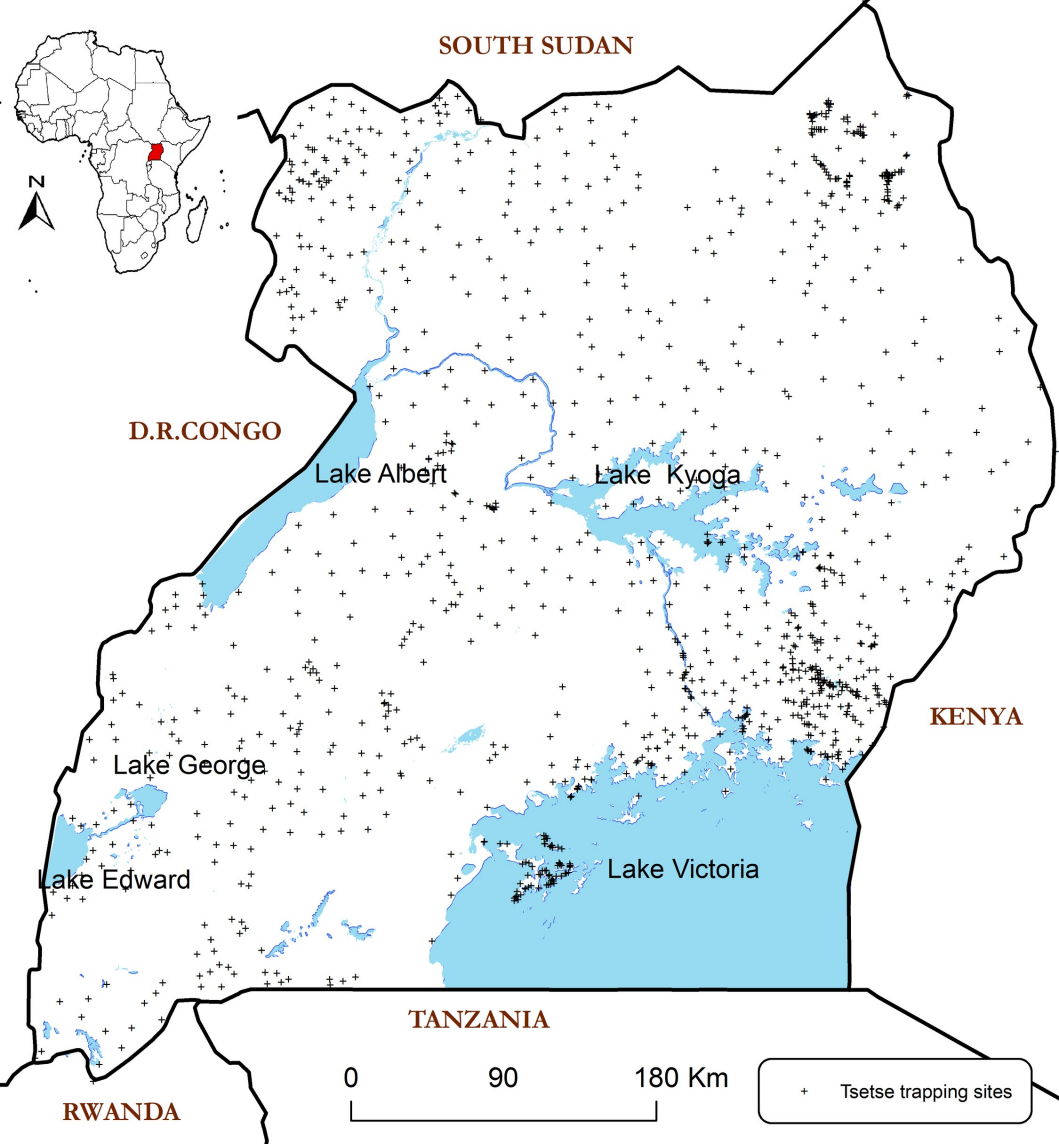
Location of areas (points) with tsetse data in Uganda as applied in the model. Base map showing country boundaries of Uganda constructed using GIS shapefiles was obtained from the public domain https://data2.unhcr.org/en/documents/details/83043. This map has been processed and visualization enabled using ArcGIS 9.1 as the GIS software.

Comprehensive national tsetse count data are difficult to obtain and, in many cases, not available. The data used in this paper were obtained from different sources grounded on a structured protocol. The Coordinating Office for Control of Trypanosomiasis in Uganda (COCTU) and Ministry of Agriculture, Animal Industry and Fisheries (MAAIF) database was the main source of data. Such data are usually obtained from district entomologists who conduct routine entomological surveillance and those that carry out baseline surveys before any well-planned tsetse control intervention is implemented. Secondly, we also used data collected through tsetse projects implemented by the central government at sub-national level. Thirdly, we used data generated by individuals or groups of researchers under research engagements. Note that some parts of the country have detailed tsetse data while in other areas data are sparse (Fig 1). We also observed and took advantage of the information on tsetse presence and abundance available in literature. This process involved a review and synthesis of several relevant tsetse and trypanosomiasis publications with tsetse survey work undertaken in Uganda between the years 2008 and 2018. Where this occurred, relevant data were extracted for use as part of candidate data for the model. This data assembly methodology was adopted from Cecchi *et al* [36] and the precise data abstraction protocol used is included as additional information (**S1 Protocol of data abstraction**).

A challenge with the candidate data is the need to integrate data collected by different groups or individuals, in different years and at different locations. To deal with this challenge, data harmonisation for the purposes of responsiveness and matching was carried out. This involved clustering data based on seasonality (dry and wet).

Fully aware that seasonal variation influences tsetse activity and survival, our focus was on dry season tsetse data which constituted the candidate data for measurement of tsetse abundance. Wet season data were less responsive during the exploratory stage. The species in focus was the *Glossina fuscipes fuscipes* (*G*. *f*. *fuscipes*), which is the dominant tsetse species in Uganda.

The major tool for tsetse capture was registered as pyramidal trap in many instances and biconical trap in others. The methods used for tsetse data collection were recorded, for example, with respect to standard operating procedures e.g., a total tsetse fly catch over 72 hours duration of each trap was almost universal for the majority of the data considered [31,32,37–39]. The model made use of tsetse catch totals for each trap site for the three days (72hrs).

### Remotely sensed variables

**Fig 2:**
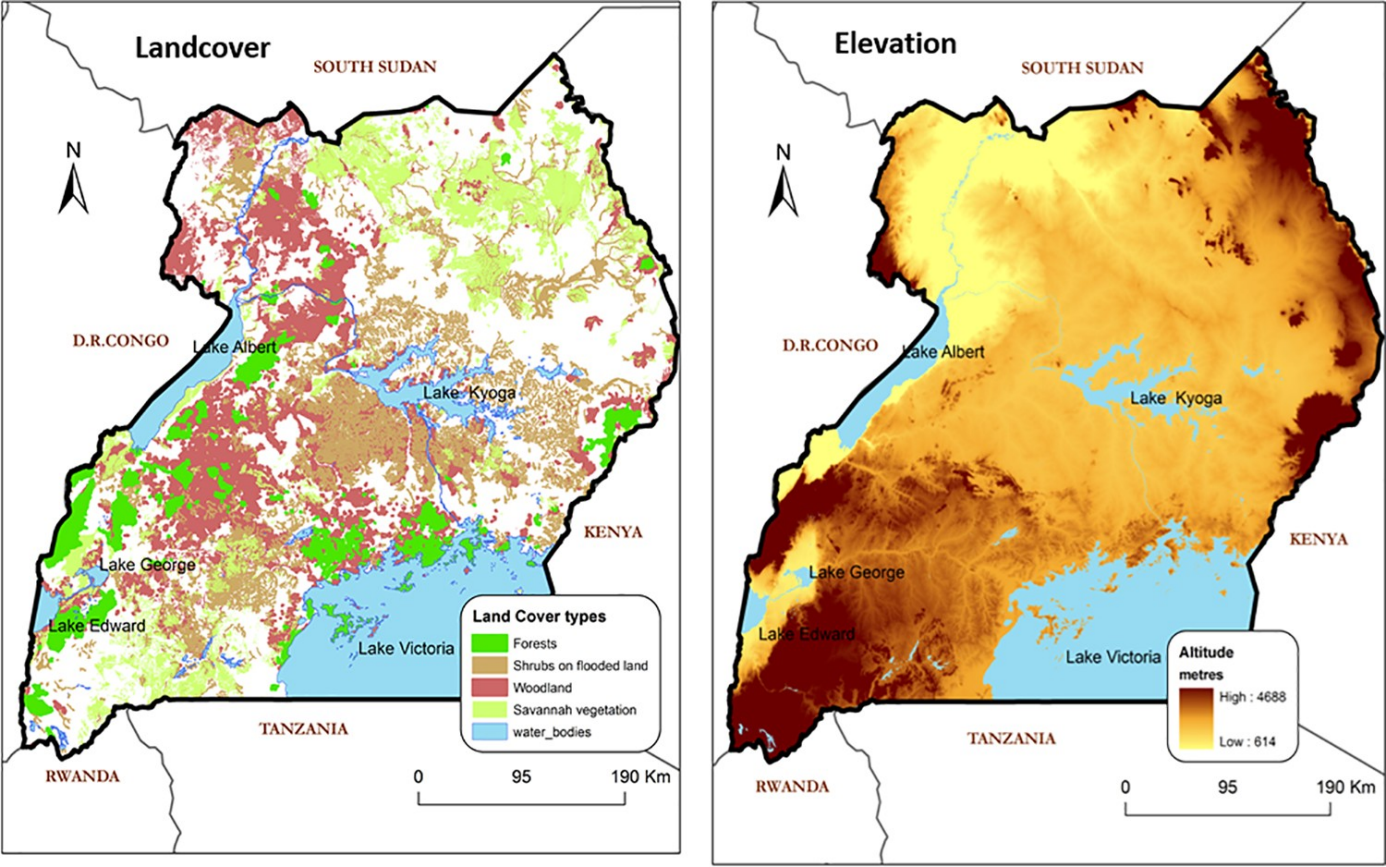
Land cover and Elevation as predictor variables used in the model. This landcover map was processed using ArGIS 9.1 based on open-source data obtained from https://data.apps.fao.org/map/catalog/srv/api/records/2a32ca87-0504-4700-8005-7a8b93974b65. While the elevation map was processed using ArcGIS 9.1 based on data from https://www.usgs.gov/core-science-systems/ngp/tnm-delivery/gis-data-download.

The covariates considered in this study include; temperature, normalised difference vegetation index (NDVI), elevation, precipitation and land cover. Land cover layer (Fig 2) is from the Africover (global land cover) and associated variables were generated from a full spatial resolution, multipurpose land cover database, produced from visual interpretation of digitally enhanced LANDSAT-Thematic Mapper (TM) images [40]. Due to spatial resolution of the land cover data used, a buffer distance of 1 Km was found appropriate and applied to each of the trap-sites to allow computation of the required parameters for use in the model. For each individual buffer (3.14Km^2^), respective areas for the various land cover types were computed in square kilometres and subsequently converted into percentages. Only ecologically influential land cover classes linked to tsetse existence [41–43] were considered. These were; natural forest, savannah vegetation, woodland, and shrub land.

**Fig 3:**
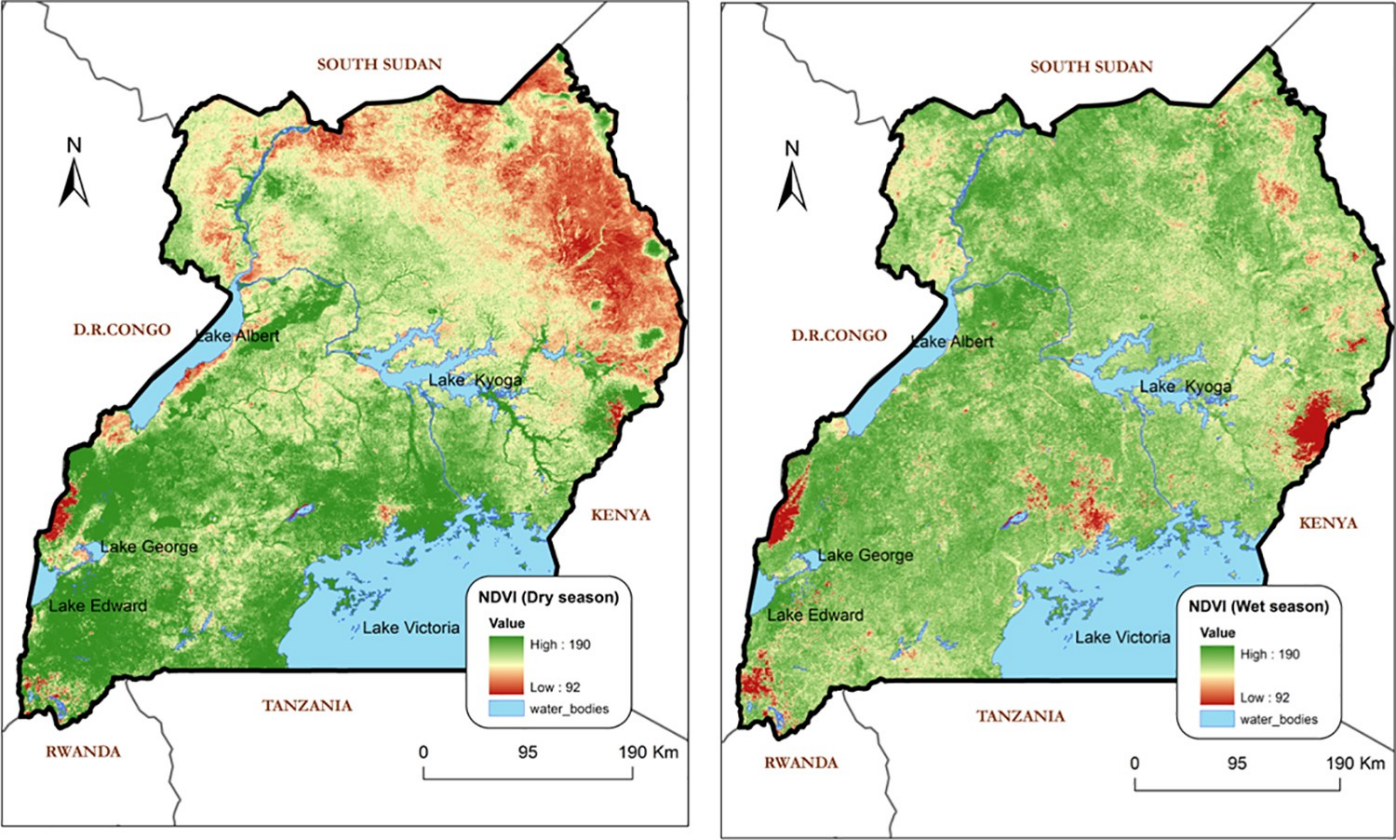
Normalised Difference Vegetation Index as predictor variable for the model. This NDVI map was processed using ArcGIS 9.1 based on data from https://fews.net/.

Normalised difference vegetation index (NDVI) (Fig 3) is a measure of vegetation cover or biomass production from multispectral satellite sensor imagery, derived from the National Oceanic and Atmospheric Administration (NOAA) satellites Global Inventory Monitoring and Modelling Studies group (GIMMS) data. Raster values of NDVI for vegetated land generally range from about 0.1 to 0.9, with values greater than 0.5 indicating dense vegetation (USGS FEWS NET Data Portal). All data are provided in GeoTIFF format with embedded colour tables. Coordinate System Description: Geographic; Units: DD (decimal degrees), Spheroid: WGS84.

**Fig 4:**
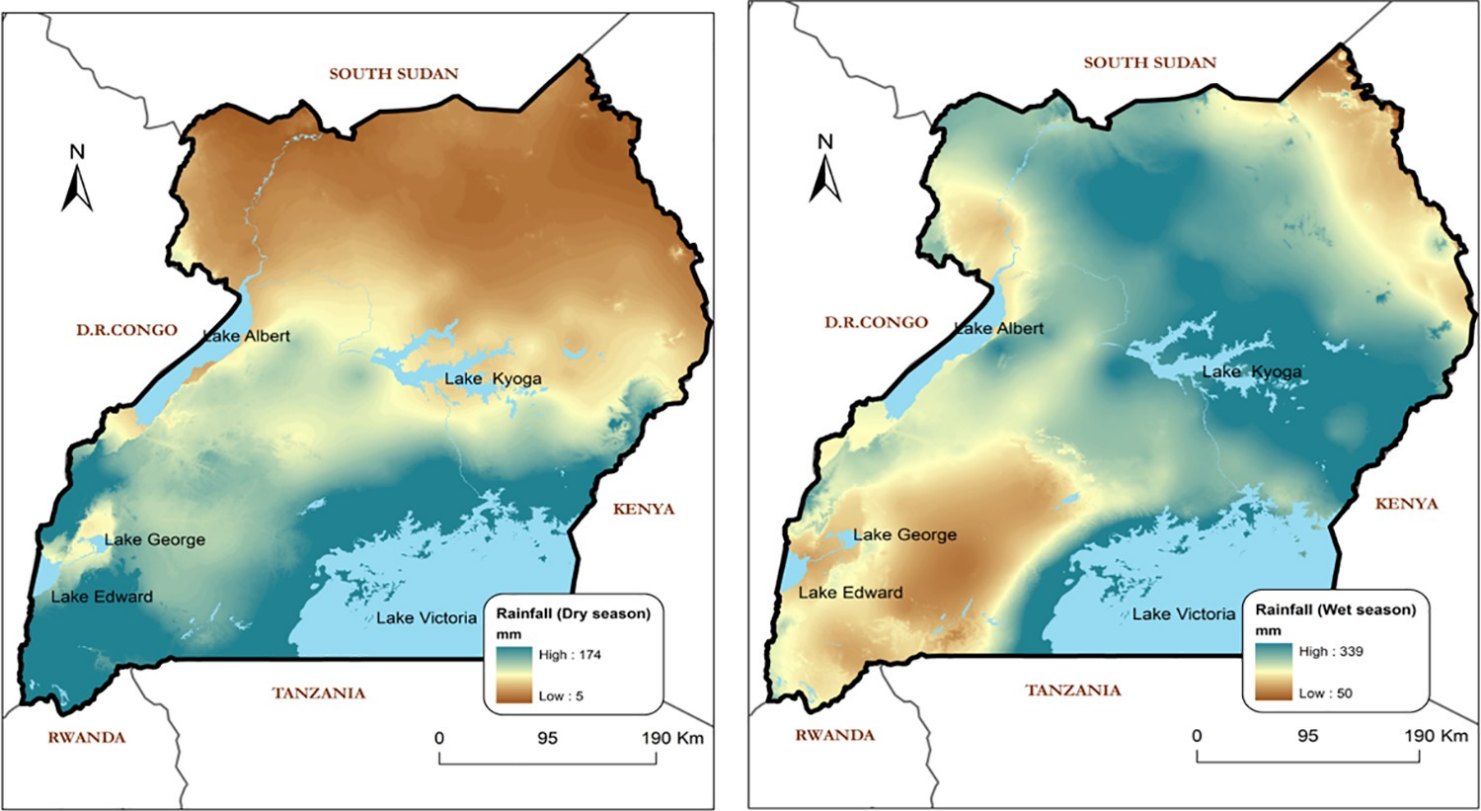
Precipitation Index as predictor variable for the model. This precipitation map was processed using ArcGIS 9.1 based on data from https://www.worldclim.org/data/worldclim21.html.

**Fig 5:**
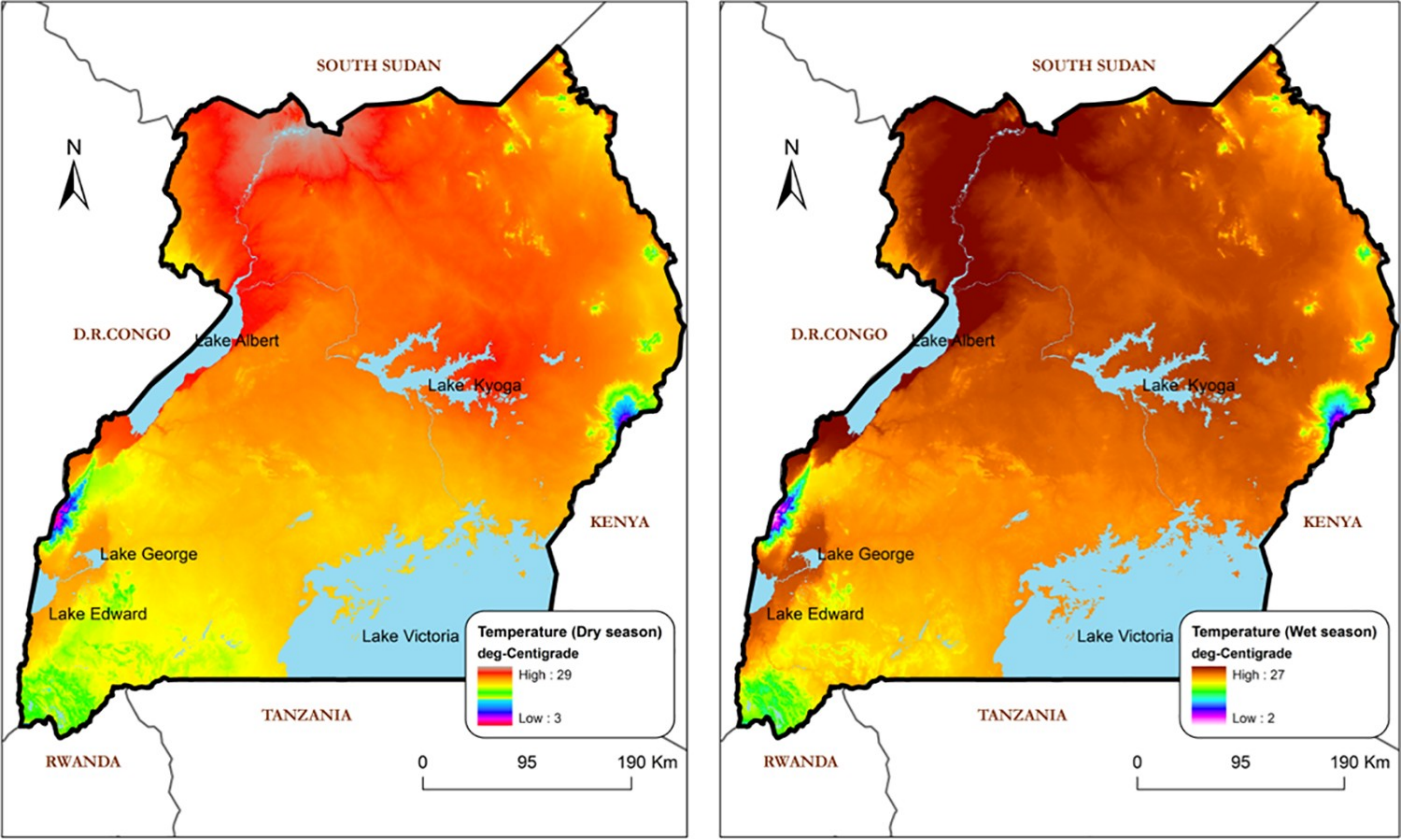
Temperature Index as predictor variable for the model. This temperature map was processed using ArcGIS 9.1 based on data obtained from https://www.worldclim.org/data/worldclim21.html.

The precipitation (Fig 4) and temperature (Fig 5) datasets used in the model were obtained as interpolated raster data and processed at a fine spatial resolution of 30 arc-secs (~1 km). The processed data were supplied by the WorldClim—Global Climate Data facility. These data were for both dry and wet season for comparison purpose. Elevation data (Fig 2) were obtained from the Shuttle Radar Topography Mission (SRTM). The SRTM is an international project spearheaded by the National Geospatial-Intelligence Agency (NGA), National Aeronautics and Space Administration (NASA), the Italian Space Agency (ASI) and the German Aerospace Centre (USGS, 2004). The elevation data were obtained and aggregated at a spatial scale of 1 km within the study area. Summary data specifications are provided in Table 1.

**Table 1:** Predictor variables used in the analyses of tsetse fly abundance including their observed maximum and minimum values in the training dataset.

Code	Name	Max value	Min value
**Meteorological data surrogates**	*Precipitation(mm)*		
	Monthly average–January 2018-dry season	137	6
	Monthly average–May 2018 –wet season	339	65
	*Temperature (* ^*0*^ *C)*		
	Temperature-Monthly average–January 2018	28.5	4.9
	Temperature-Monthly average–May 2018	28.2	4.6
**Vegetation surrogates**	*Normalised difference vegetation index (NDVI)*	*Raster values*	
	NDVI-1- January 2018 dry season	188	94
	NDVI-2 –May 2018 wet season	190	92
**Altitude**	*Elevation (m)*	4427	615
**Land cover**	*Land cover* (09 Classes)	n/a	
**Game Parks**	Game parks distribution layer		
**Tsetse dry season data**	Apparent trap catch	576	0

*The final model makes use of dry season environmental and climatic variables. These are the variables that influence and determine tsetse abundance.

### Statistical analysis

**Fig 6:**
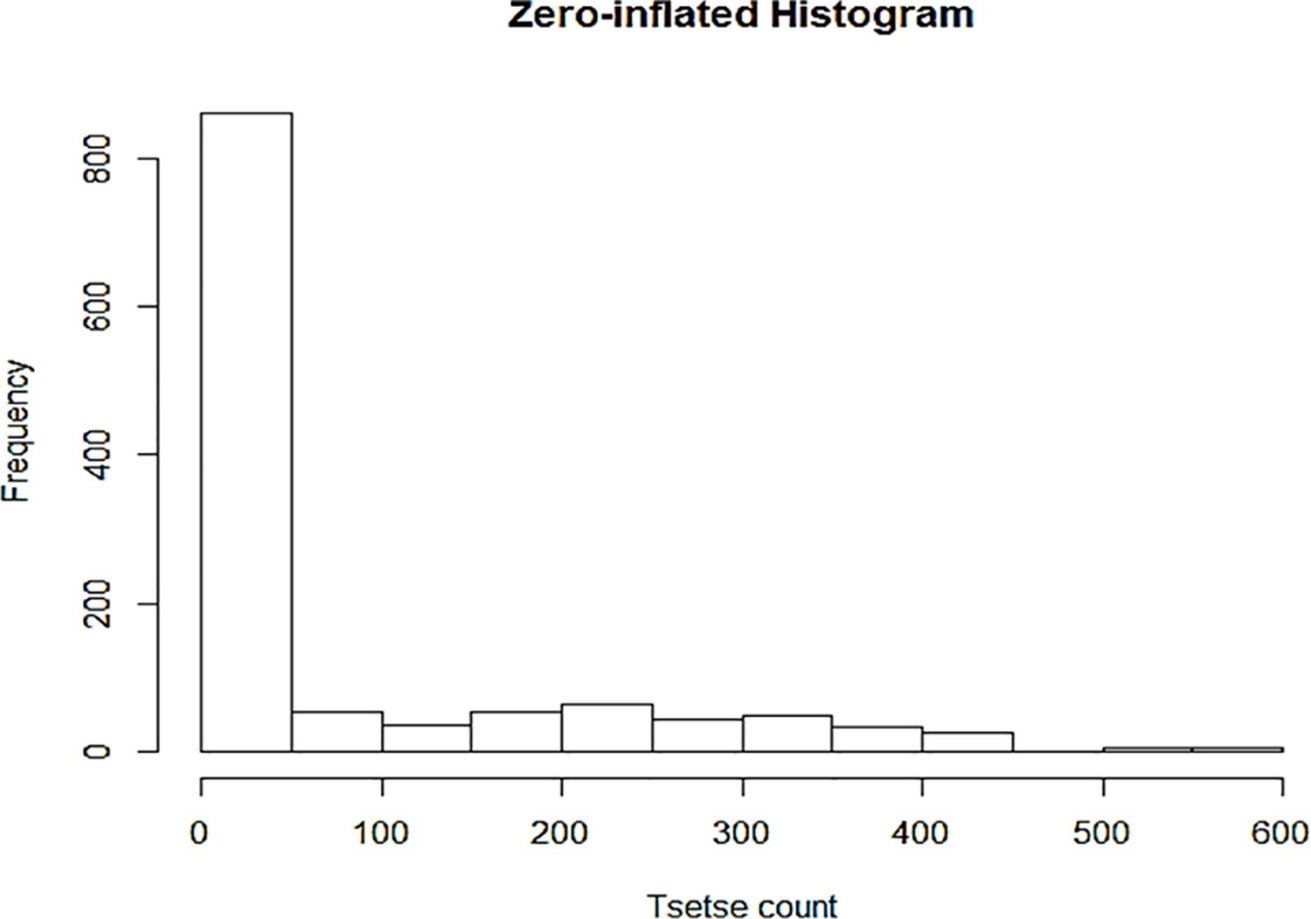
Frequency histogram showing number of tsetse flies at all locations in Uganda–a case of zero-inflation and over-dispersion. This is an output of the tsetse data exploration performed using R-tool.

Two methods commonly considered for statistical analysis of count data were adopted. These are Poisson regression and Zero-Inflated Poisson regression [44]. The choice of method was dictated by the data characteristics. The abundance variable is essentially a count variable and, in this case, contained many zeros and clear outliers accounting for apparent overdispersion (Fig 6). Thus, two regression models were fitted (i.e the standard Poisson regression and Zero-Inflated Poisson regression). The study made use of ‘weights by inverse distance’ as the distance–weighting scheme in the final mapping within the ArcGIS facility.

Exploratory analysis was undertaken to guide model selection. It involved the construction of; (i) scatterplots (to test for collinearity), (ii) boxplots / dot-plots (to identify outliers), (iii) correlation matrix (to test for multicollinearity) and (iv) histograms / QQ-plots (to test for normality) within the datasets [45,46].

Fitness of the Poisson and Zero-Inflated Poisson regression models was assessed through; (i) computation and examination of the deviance as an approximate goodness-of-fit test, (ii) comparison of residual deviance with the *χ*^2^ distribution, (iii) examination of the Akaike information criterion (AIC) score and (iv) generating residual plots. The standardised deviance residuals were approximately normally distributed with equal variance if the model assumptions were satisfied [18,45–47]. Further, the correlation between observed and predicted values was computed. Model outputs were evaluated against each other to confirm if the ZIP model outperformed the standard Poisson regression in tsetse abundance estimation. This was done by performing a Vuong test of the two models [48]. Spatial prediction of tsetse abundance in the country was carried out using the final multivariate Zero-Inflated Poisson model parameters.

### Model specifications

The Poisson regression model expresses the log of the observed count as a linear function of a set of predictors, such that; log_e_ (*Y*) = *β*_0_ + *β*_1_*Χ*_1_ + *β*_2_*Χ*_2_ + *εχ*_0_ and Y = (*e*^β^_0_) (*e*^β^_1_^Χ^_1_) (*e*^β^_2_^Χ^_2_) *etc* [44,46,49,50]. Consider **Y** = Tsetse fly number (variable to be predicted by the model), *β*_0_ = intercept / estimated constant (also taken as ln(n)), *β*_*i*_ = computed coefficients for each explanatory variable and *Χ*_*i*_ = explanatory variable (model can be extended to include multiple predictors).

The dependent variable is a count of the occurrences of interest; in this case the tsetse fly numbers identified in a geographical location. This method enables one to estimate an Incidence Risk Ratio (IRR) associated with a given predictor or exposure [46]. This ratio is important as it informs of incremental changes in the outcome variable (tsetse abundance) due to variations in predictor variables or covariates. To determine the appropriateness of Poisson regression, a histogram for tsetse count (Fig 6) was constructed and the mean, variance, standard deviation and deviance computed. The shape of the outcome histogram guides on the relevance of applying the Poisson regression on the dataset.

The second stage of data analysis was based on the *Zero-Inflated Poisson regression*. This regression is considered as a generalization of Poisson regression as it has the same mean structure as Poisson regression but with an extra parameter to account for the over-dispersion. It is a modification of the standard Poisson regression model to allow for an over-abundance of zero counts in the data [44,46]. Failure to address the over-dispersion condition usually leads to underestimation of standard errors causing incorrect assessment of the significance of individual regression parameters. The essential idea is that the data come from two regimes. In one regime (*R*_I_) the outcome is always a zero count, while in the other regime (*R*_II_) the counts follow a standard Poisson process [46].

Preliminary visualisation of the point-based tsetse survey data and extraction of covariate values needed for analysis was enabled through the use of the ArcMap10 GIS software. Exploratory analysis, that is, univariate and multivariate analyses, were performed using the R statistical software–Zanzibar version (R Development Core Team 2019). In this study, the two approaches were assessed statistically and the best fitting model used in the final prediction of tsetse fly abundance for the study area.

### Univariate parameter estimation for Poisson regression

For both regression models, all covariates were assessed individually against the tsetse count data for responsiveness. As a set condition, all covariates with a *p*-value greater than 0.05 were excluded from further analysis. Significant variables in the univariate analysis were further subjected to cluster correlation and a correlation matrix generated to detect any aspects of multi-collinearity. Where this occurred, it was decided to remove the least significant covariates from further analysis.

### Multivariate parameter estimation for Poisson regression

Significant predictors delivered from the univariate analysis stage (p<0.05) were combined in a multivariate regression analysis to find the best fitting model in each case. A forward-step-wise approach was applied to enable exclusion of next level non-responsive variables from the model, resulting in a final multivariate regression model. Covariates were added starting with the most significant; cumulatively one after the other. Each covariate had to maintain its statistical significance (*p*-value < 0.05) to be retained. Estimated model coefficients were compared with those obtained at the univariate analysis stage to ascertain the consistency of final covariates in influencing the outcome variable. To check for spatial autocorrelation in the residuals from the final model, a residual variogram [46,51] was constructed. The process enabled computation of the coefficients, Incidence Risk Ratio (IRR), standard errors, probability values and the confidence interval for the standard Poisson regression model [46].

### Multivariate parameter estimation for Zero-Inflated Poisson (ZIP) regression

A ZIP model was fitted based on the final standard multivariate Poisson regression model outcomes. The ZIP model has two parts, a Poisson count model and the (Zero) logit model for predicting excess zeros [46,48] This is intended to account for the excess zeros with the understanding that these are generated by a separate process from the count values and that the excess zeros can be modelled independently. Similarly, the process enabled computation of the coefficients, Odds Ratio (OR), standard errors, probability values and the confidence interval for the Zero-Inflated Poisson model. Finally, the two models were compared against each other using the Vuong test-statistic which is asymptotically distributed as ~*N*(0,1) under the null hypothesis that the models are indistinguishable [46,48]

## Results

**Fig 7:**
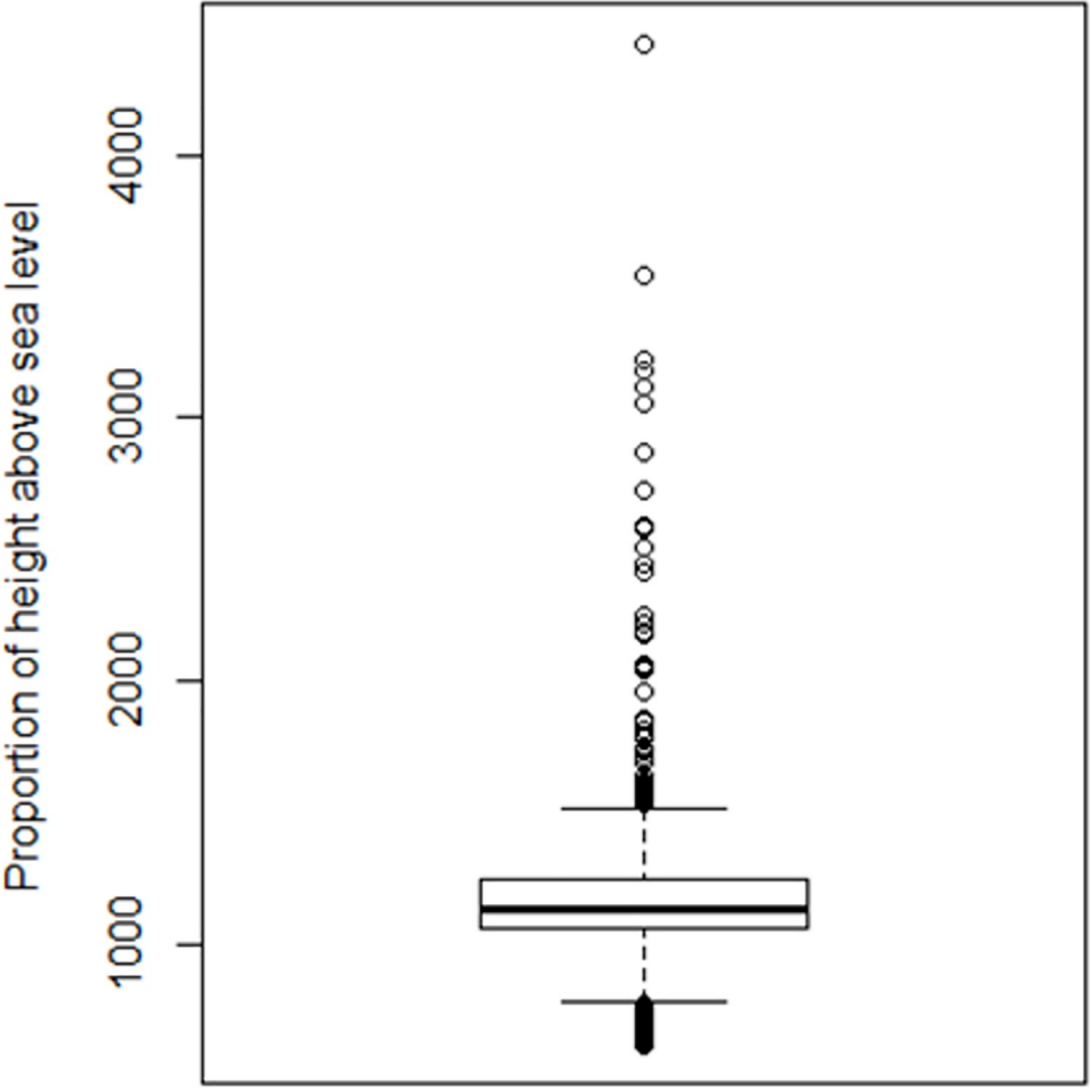
Exploratory analysis of elevation. In terms of altitude, the boxplot shows that the majority of tsetse points were recorded within 1100 and 1500m above sea level. One point lies above 4000m and that is a typical outlier. These points do not show the same relationships as the bulk of the data. Therefore, it was decided to eliminate such extreme outliers in all cases.

**Fig 8:**
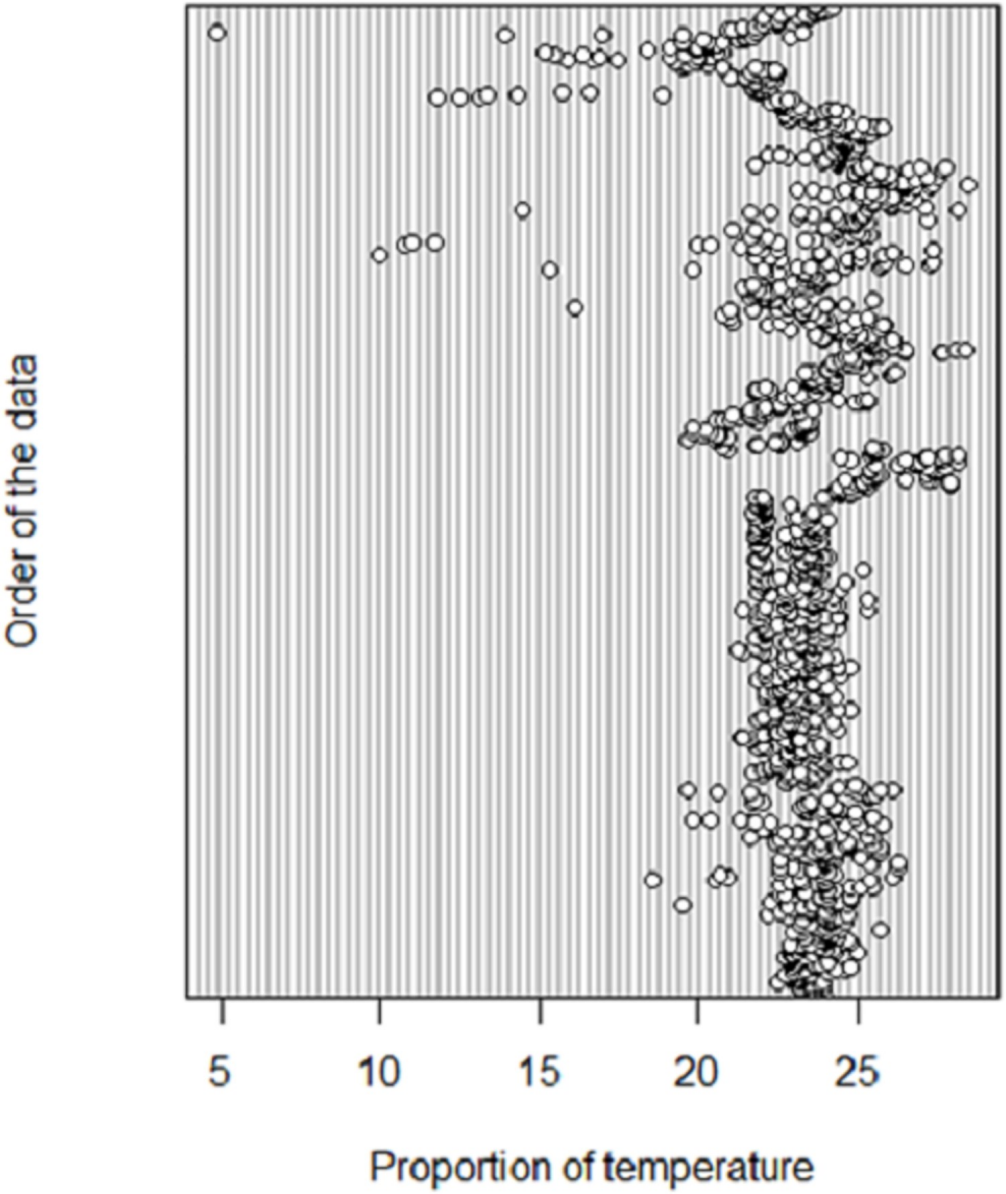
Exploratory analysis of temperature. In terms of temperature, the dot-chart shows that the majority of points lie between 23°C and 26°C with a few outliers at 6°C. Therefore, it was decided to eliminate such extreme outliers in all temperature figures or cases.

During exploratory analysis, several plots and charts were generated. An example of two plots is given in Figs 7 and 8, which enable visualisation of the tsetse data in relation to elevation and temperature using box-plots and dot-charts, respectively [3,17]. These plots reveal the existence of outliers in the candidate data.

Table 2 lists the estimated predictor variable coefficients with their associated statistics for the multivariate *Poisson* analysis. All covariates were found to be significantly associated with the exploratory variable at *p*<0.05 [45,46]. Association for six of these studied covariates were positively significant (IRR>1) and the rest were negative. Significance of the association of Woodland, Elevation and Temperature were negative (IRR<1). The results from the standard Poisson model indicate that tsetse abundance is largest at low elevations, in areas of high vegetative activity, in game parks, in forests and in permanently flooded shrubs.

**Table 2:** Multivariate Poisson regression coefficients and their *p*-values, IRR and confidence intervals.

Predictor variable	Est.Coefficient	P_Value	IRR	95%CI
Intercept	14.5539	P<0.01	2.092720e+06	1.5e+06–2.9e+06
Natural Forest**	0.1509	P<0.01	1.1629	1.1174–1.2103
Savannah**	-0.0867	P<0.01	3.3730	3.3439–3.4023
Shrubs on flooded land**	0.2721	P<0.01	1.3128	1.3038–1.3218
Woodland**	-5.0189	P<0.01	0.3291	0.2992–0.3618
Elevation	-2.1852	P<0.01	0.4136	0.3894–0.4394
NDVI*	0.6219	P<0.01	1.8625	1.7708–1.9591
Rainfall	-0.1933	P<0.01	3.0320	3.0182–3.0460
Temperature	-3.2549	P<0.01	0.5221	0.4724–0.5770
Game Parks	-0.3151	P<0.01	2.6842	2.6025–2.7683

*NDVI: Normalised Difference Vegetation Index

**The parameters used against each landcover variable were entirely computed as a percentage of composition in the 1km buffer drawn at each trap site.

In one regime (*R*_I_) the outcome is always a zero-count, while in the other regime (*R*_II_) the counts follow a standard Poisson process. Under Regime (*R*_II_), game parks with statistics of *p* = 0.489, IRR = 2.365 and a coefficient of 0.011 appear to be insignificantly associated with tsetse abundance. The rest of the predictors are strongly associated with tsetse abundance (p<0.01) with different magnitudes. Table 3 illustrates the superior fit of the ZIP model over the Poisson model. The test statistic is significant, indicating that the zero-inflated model is superior to the standard Poisson model.

**Table 3:** Zero-Inflated Poisson regression results for estimation of tsetse abundance based on climatic and environmental factors. Below are two blocks (regimes); one is a block of output containing Poisson regression coefficients for each of the variables along with *p*-values, incident risk ratios and confidence intervals for the coefficients. The second block includes logit coefficients for predicting excess zeros along with their Odds Ratios (OR), confidence intervals and *p*-values.

**Predictor variable** ^**(1)**^	**EST.Coefficient**	**P_Value**	**IRR**	**95%CI**
Natural Forest	0.404	P<0.01***	1.499	1.437–1.563
Savannah	- 0.044	P<0.01***	3.519	3.486–3.551
Shrubs on flooded land	0.327	P<0.01***	1.387	1.377–1.397
Woodland	- 3.267	P<0.01***	0.516	0.470–0.566
Elevation	- 2.749	P<0.01***	0.866	0.815–0.919
NDVI	1.002	P<0.01***	2.723	2.584–2.870
Rainfall	- 0.277	P<0.01***	2.790	2.776–2.803
Temperature	- 5.204	P<0.01***	0.273	0.246–0.304
Game Parks	0.011	0.489	2.365	3.605–1.043
**Predictor variable** ^**(2)**^	**Est.Coefficient**	**P_Value**	**OR**	**95%CI**
Intercept	12.1380	0.0023 **	1.86e+05	7.57e+01–4.60e+08
Natural Forest	0.77174	0.0500*	2.1635	3.6727–4.6886
Savannah	-0.00973	0.9080	3.6431	1.1681–3.0885
Shrubs on flooded land	0.06263	0.5141	1.0646	1.2850–3.2448
Woodland	3.79927	1.65e-08 ***	12.1422	3.2467–12.3435
Elevation	-1.71091	0.0144 *	0.6647	0.6208–2.6186
NDVI	1.28711	0.0393 *	3.6223	1.0647–3.3496
Rainfall	-0.32996	7.38e-08 ***	2.6448	2.3454–2.9825
Temperature	-5.44601	3.43e-06 ***	0.2147	0.0792–0.5816
Game Parks	0.86096	0.0097 **	2.3654	1.2313–4.5439

^(1)^ Regime (R_II_): *Count model coefficients (Poisson with log link)*

^(2)^ Regime (R_I_): *Zero-inflation model coefficients (binomial with logit link)*

* p-value <0.05

** p-value <0.01

*** p-value<0.001

The ZIP statistics indicate that natural forests, woodlands, NDVI, Rainfall and game parks positively influence tsetse abundance. Under regime *R*_I_, savannah vegetation and shrubs on flooded land loose their significance as *p*-value is greater than 0.05 (Table 3). Temperature and elevation inversely impact on tsetse abundance (OR<1). This analysis shows that the major drivers of tsetse abundance across Uganda are natural forests, woodlands, NDVI, Rainfall and game parks. From the data used, monthly rainfall amounts varied between 6 mm and 339 mm for the different parts of the study area. The low rainfall in the dry season had a marked, significant association with tsetse abundance in the univariate analysis. Actually, both Temperature and Rainfall had no effect on tsetse abundance during the wet season. But again rainfall became key and significant in the meaningful measurement of tsetse abundance based on the dry season data.

**Fig 9:**
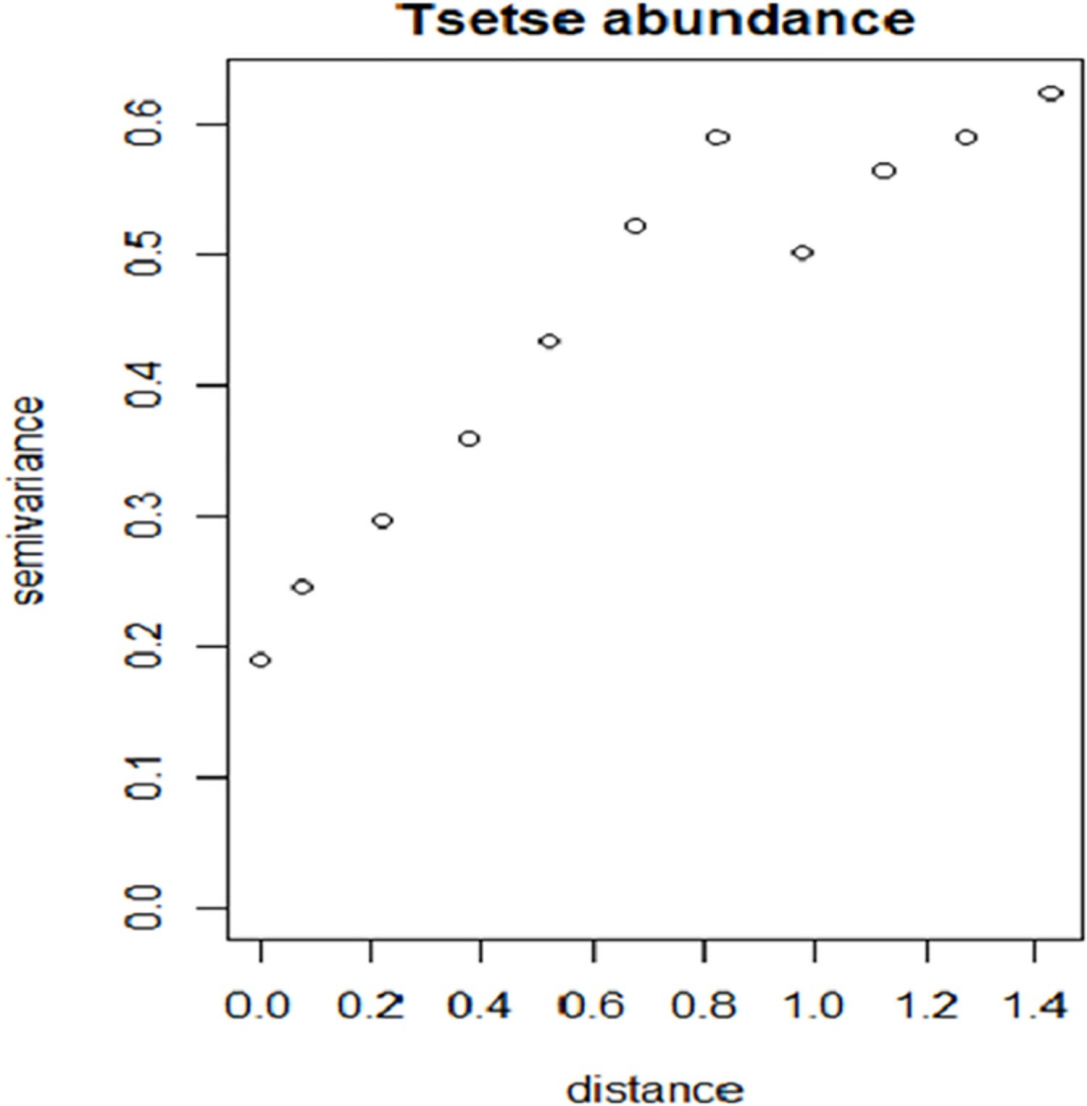
Residual variogram of the Pearson residuals based on the Poisson regression model (reveals the existence of spatial autocorrelation within the residuals). This residual variogram is an output of model fitting using R-tool under ordinary Poisson regression.

The empirical variogram of the Pearson’s residuals [45,46] in the Poisson regression model in Fig 9 indicates the presence of non-explained spatial variation in the residuals. This shows that the model residuals are not independent and thus invalidates the model in a formal sense.

**Fig 10:**
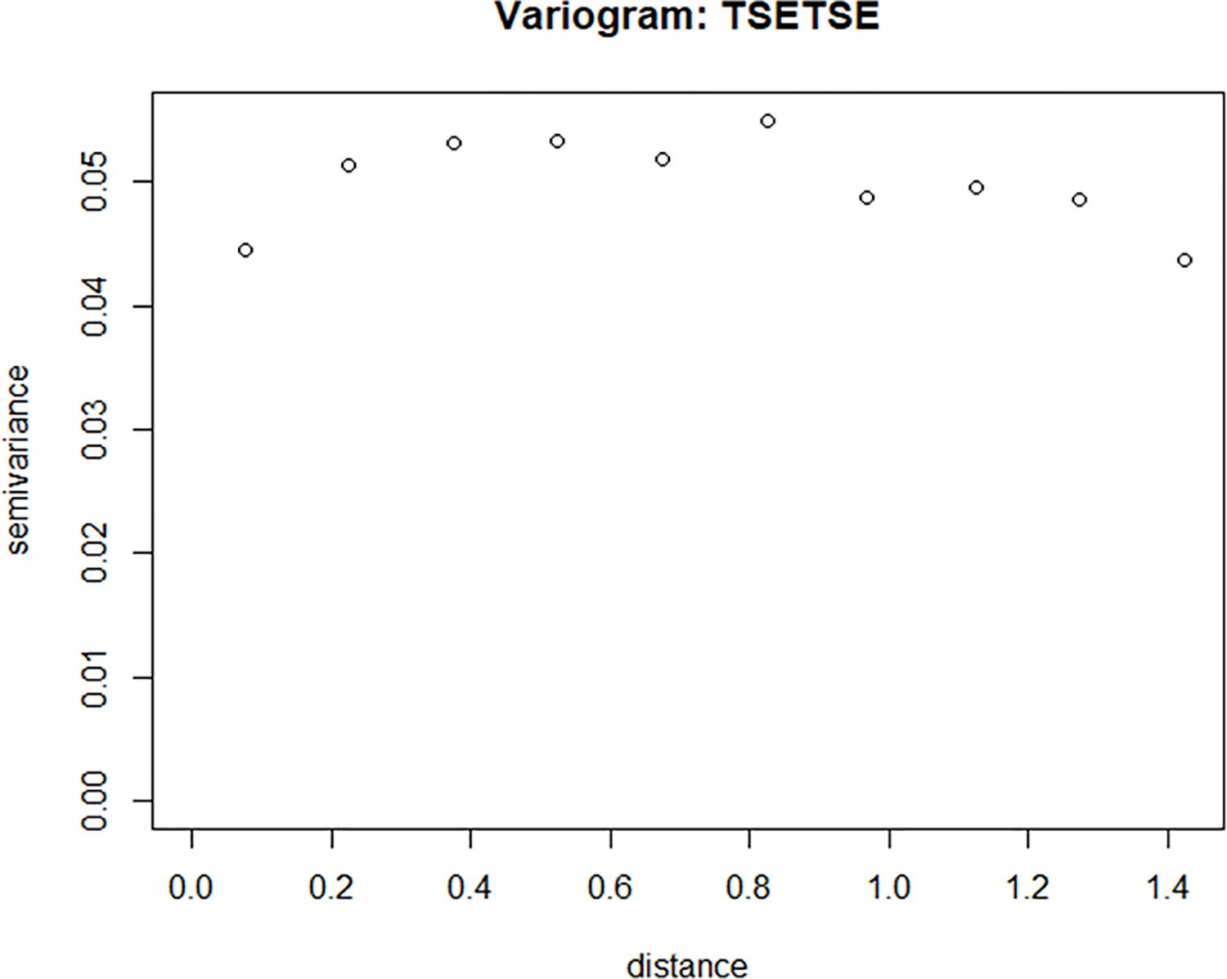
Residual variogram of the Pearson residuals based on the ZIP regression model (largely reveals the spatial independence of the residuals). This Residual variogram is an output from model fitting using R-tool under Zero-inflated Poisson regression

Residual spatial autocorrelation from the ZIP regression model is much less evident (Fig 10). This provides confidence that the ZIP model has, to some extent, accounted for the spatially correlated variation in tsetse count data.

**Fig 11:**
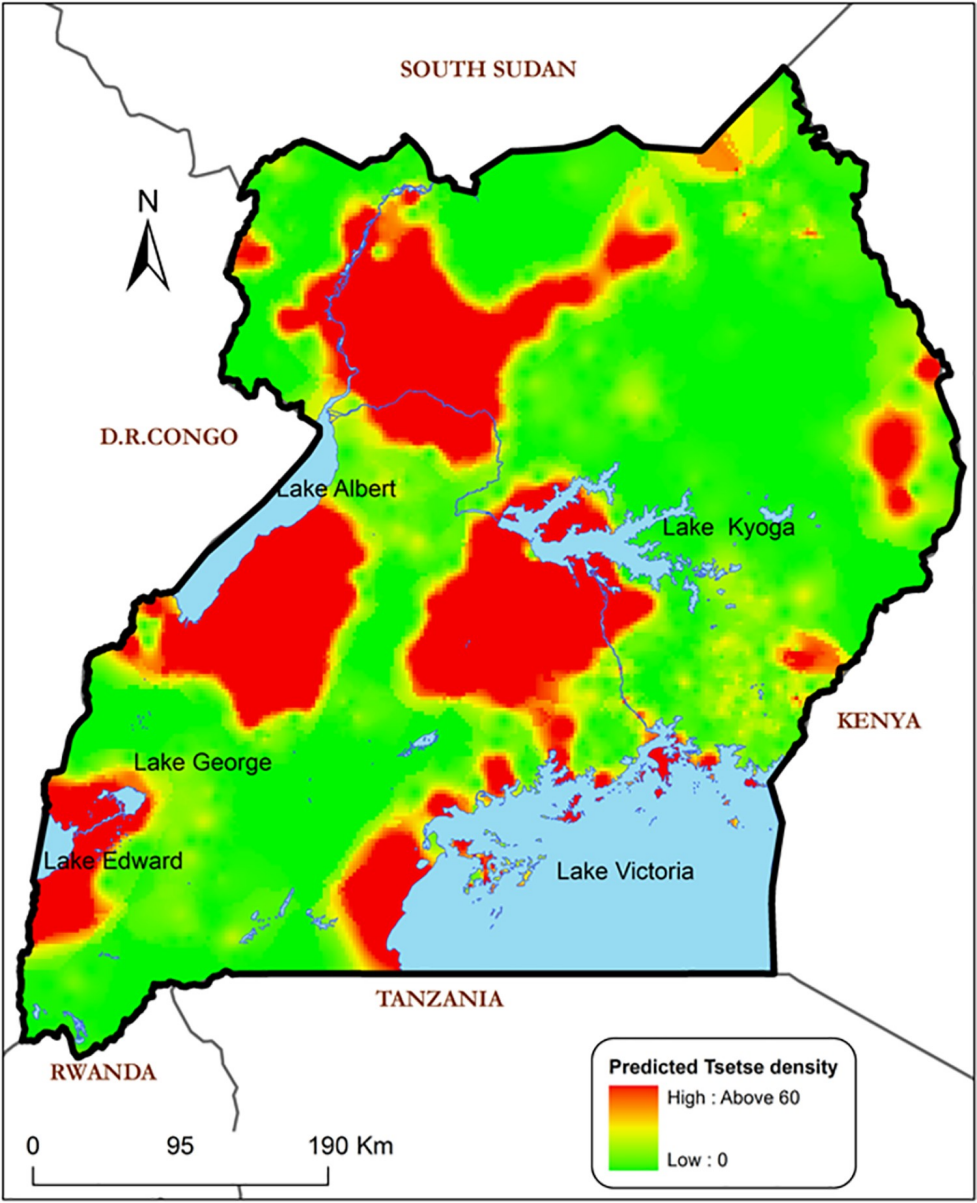
Predicted tsetse fly (Gff) abundance using the ZIP model. This tsetse prediction map is an output of the Zero-inflated Poisson regression model using R-tool and ArcGIS 9.1. Base map showing country boundaries of Uganda processed from GIS shapefiles obtained from the public domains https://data2.unhcr.org/en/documents/details/83043.

Fig 11 shows the predicted tsetse abundance based on the final ZIP regression model. The model identifies areas of scaled tsetse abundance with estimated apparent fly densities ranging from 0 to 58 tsetse flies. The predicted abundance map reveals some isolated areas which are thought to be tsetse free zones to return high apparent tsetse densities and *vice versa*. Higher predicted abundances of *G*.*f*. *fuscipes* tsetse flies were observed in areas around all game reserves (Kidepo, Murchison, and Queen Elizabeth), all the Lake Victoria shores including its islands, parts of Lake Kioga and Lake Albert shores.

This output illustrates the tsetse extent as categorised into low, medium and high with particular reference to tsetse constrained and non-constrained populations

## Discussion

To predict abundance of *G*. *f*. *fuscipes* across Uganda, a total of nine predictor variables were investigated in two models, standard Poisson, and ZIP. The standard Poisson model indicated that all nine predictors were associated with tsetse abundance. The ZIP model demonstrated that only six predictors (i.e. rainfall, temperature, game parks, NDVI, elevation and woodland) strongly influenced tsetse abundance. Natural forests and shrubs on flooded land were both not associated with the response variable. Savannah vegetation returned a statistically insignificant association. Specifically, only six predictors highly and positively influenced tsetse abundance. Two covariates showed a negative influence on tsetse abundance. These were; temperature and elevation.

*G*. *f*. *fuscipes* abundance was positively correlated with the presence of shrubs along the flooded land. This class of land cover consists of shrub vegetation on permanently or temporarily flooded land (crown cover between 15 and 100%); the height is in the range of 0.3–5 m. This class occupies more than 4% of the total surface of Uganda (10,000 km^2^) [41,42,52–54]. The humid environment and the shading provided by the shrub vegetation can provide a suitable habitat for many tsetse species. This is supported by the model results. The model identifies this land cover class as being influential and it indicates that tsetse abundance increases proportionate (OR = 1.065) to the increase in percentage of shrubs existing on flooded land. The near permanent interaction between shrub vegetation and water provides ideal survival conditions for *G*. *f*. *fuscipes*.

**Fig 12:**
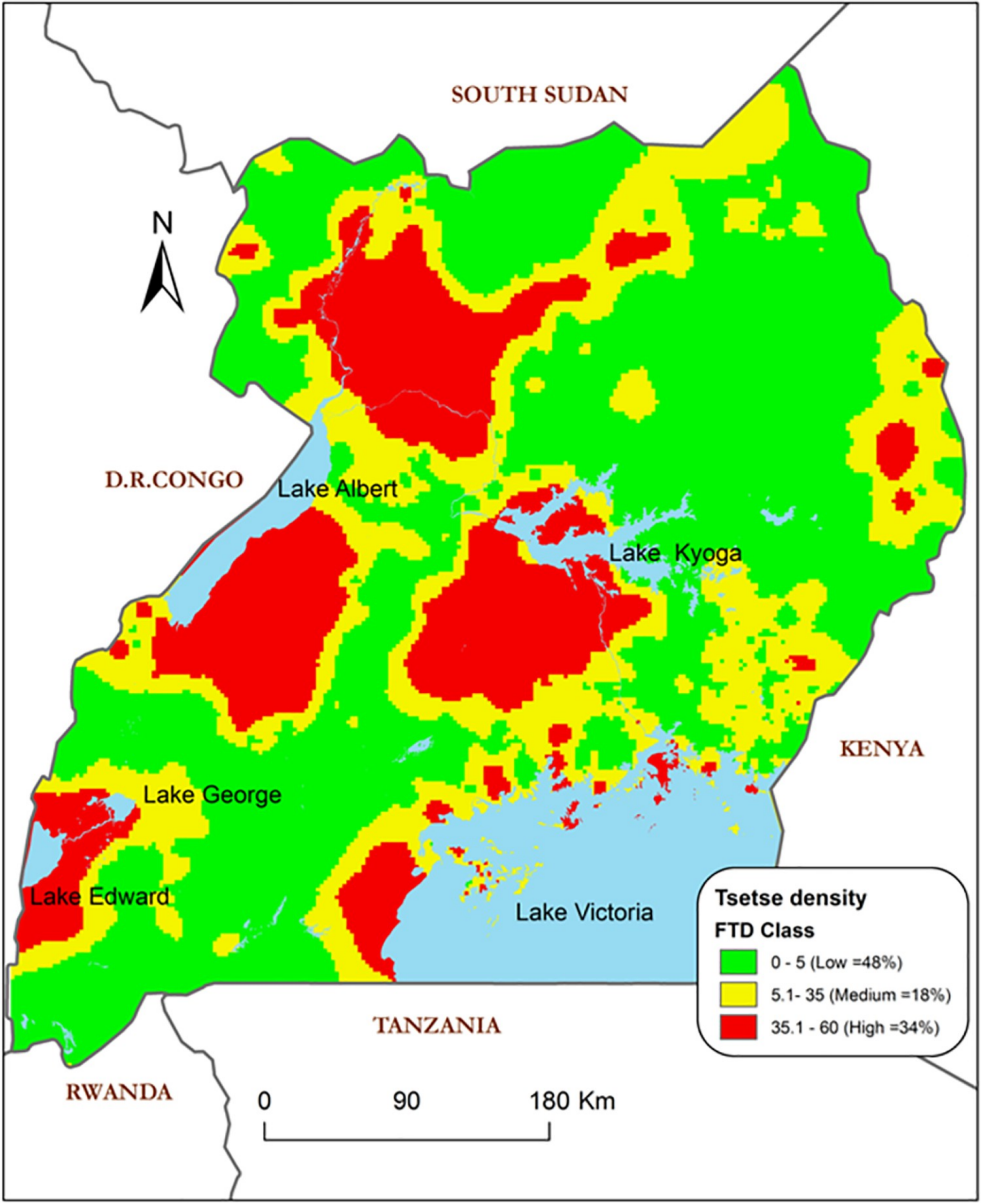
Predicted tsetse fly (Gff) abundance using the ZIP model categorized in three classes. Base map showing country boundaries of Uganda processed from GIS shapefiles obtained from the public domains https://data2.unhcr.org/en/documents/details/83043.

Elevation influences the micro-climatic conditions of an area. The entire study area has significant height variation (615 to 4427 m above sea level) and the ZIP model illustrates an altitudinal control on tsetse abundance within the study area (*p* = 0.0144, OR = 0.6647). Altitudinal control is illustrated by an odds ratio (OR) below one. Tsetse abundance is perceived to increase with declining altitude at a rate of 0.66. Areas at significantly high altitudes (above 3000 m asl) have their tsetse abundance rated as zero or at least closest to zero. Such areas are clearly observed in Kigezi highlands, Rwenzori and Elgon mountains (Fig 12) among others. The model illustrates that tsetse abundance increases steadily as altitude decreases. Similarly, areas below 1000 m asl do not favour tsetse existence and are, thus, marked with low tsetse abundance by the model. Altitude influences surface temperature and rainfall. Lowlands have drier environmental conditions with low humidity while highlands are cooler with high humidity: both extremes constrain the life of the tsetse fly [19,55].

Tsetse flies thrive in areas with mean annual temperatures of 19–30°C [17,19]. Temperatures below 19°C slow down tsetse activity and general physiology [19], while extreme temperatures increase fly mortality [19,55,56]. Tsetse are severely affected by high temperature conditions and once exposed to a temperature of more than 36°C, tsetse will have a survival capacity of close to zero [19,33]. According to the data used, the lowest temperature was registered as 6°C, the mean temperature was 24°C and the maximum temperature recorded was 28.5°C for the study area. Temperature variation was by about 23°C at most across the region. The model illustrates the nature of the association between dry season temperature and tsetse abundance. That is, tsetse infestation increases with a comparable decline in temperature (*p*<0.001, OR = 0.2147) during the dry season. These temperature extents have specific consequences on fly abundance in the study area as they do affect tsetse fly activity, general physiology and survival.

Surrogates of vegetation and meteorological data have been correlated with vector abundance and even vector mortality [14,57]. In the majority of previous tsetse distribution and abundance models, temperature emerges as the most important predictor followed by NDVI and then precipitation [14]. These findings are supported by this study. NDVI captured in the dry season has a positive correlation with tsetse abundance (*p* = 0.0393, OR = 3.622, 95%CI = 1.0647 to 3.3496). The model statistics indicate that tsetse abundance increases proportionate to increases in NDVI levels (green vegetative cover) in the dry season. Thus, during the dry season, high abundance is expected in areas where there is substantial vegetation cover and *vice versa*.

The model identifies areas with high rainfall and matches them with high tsetse abundance. The rate of incremental change in abundance is 2.6448 per degree as illustrated by the corresponding odds ratio. During the dry season, rainfall is greatly limited and any chance of heavy rains will influence environmental conditions (humidity, temperature, air circulation etc.) positively for the tsetse fly. Therefore, most tsetse flies will be traced within or very close to areas receiving most rainfall with fewer flies or no flies at all in areas with the least rainfall in the dry season.

Woodland is a class of land cover characterised by open trees with crown cover of between 10 and 70% and height ranging between 3 to 30 m or more. The vegetation is spread over the occupied area without intervals or breaks. In Uganda, this class of land cover occupies close to 4000 km^2^ (i.e 6% of the total surface of the country). Woodlands are typical habitats of tsetse flies [41,42,53,58]. Woodlands are favourite habitats for all the three major groups of tsetse (i.e *morsitans*, *palpalis* and *fusca* groups). This fact is validated by the ZIP model (Table 4) as it conveys this implicit suitability. The model illustrates the strongest association of woodland with tsetse abundance (*p*<0.001, OR = 12.1422, 95%CI = 3.2467 to 12.3435) among all predictors. The model shows that tsetse abundance increases considerably with increasing proportion of woodland as a land cover.

**Table 4:** Vuong Non-Nested Hypothesis Test-Statistic: comparison of ZIP model against the standard Poisson model. This Test-statistic is asymptotically distributed N(0,1) under the null that the models are indistinguishable.

	Vuong z-statistic	H_A	P-value
**Raw**	**-15.21836**	**model2 (ZIP) > model1(Poisson)**	< 2.22e-16 ***
**AIC-corrected**	**-15.21325**	**model2 (ZIP) > model1(Poisson)**	< 2.22e-16 ***
**BIC-corrected**	**-15.20016**	**model2 (ZIP) > model1(Poisson)**	< 2.22e-16 ***

In Uganda, game park s occupy close to 40% of the land mass. This class of predictor overlaps with some of the already listed predictors. It contains national game parks, game reserves and gazetted hunting areas. The advantage with this predictor is that it carries with it essential natural attributes as a tsetse predictor [58]. It has wild game and natural environmental settings. These zones have also benefited from zero (or limited) human interference. It is generally acknowledged that game parks are heavily tsetse-infested in Uganda. Equally, the model identifies a strong positive association between game parks and tsetse abundance and henceforth categorises it as a key predictor. The model statistics (*p* = 0.0097, OR = 2.3654, 95%CI = 1.2313 to 4.5439) indicate that tsetse abundance increases with game park presence. Our results designate the gradual reduction in tsetse apparent densities away from the game parks due to declining intensity of savannah vegetation. Similar responses particularly for *G*. *morsitans* have been reported in Tanzania and Malawi [58].

Tsetse abundance increased with the presence of a significant interaction between natural forest cover and water environments. Tsetse, especially *G*.*f*.*fuscipes*, thrives in environmental conditions where the vegetation is not too dense such as to enable them fly easily and to be able to spot the feeding host readily in some situations [8]. Above all, *G*.*f*.*fuscipes* is ecologically considered as a riverine species commonly found in zones of high humidity offered by the interaction between forest vegetation and water bodies [14,41,42,49,53]. Thus, this finding supports existing entomological understanding of the *G*.*f*.*fuscipes* vector. However, the low spatial resolution of the land cover layer which served as the source of the forest cover was unable to allow detection of very small rivers and water features associated with forest cover. This consequently affected the model results for forest influence as very fine spatial resolution effects could not be realised. Despite this, the model illustrates that tsetse abundance increases with an increase in the proportion of natural forest cover (*p* = 0.0504, OR = 2.1635, 95%CI = 3.6727 to 4.6886). The model also picks up the riverine and lakeshore forest vegetation leading to predicted high tsetse abundance along river systems and the lakeshores of Victoria and Kyoga.

Many traditionally known tsetse habitats have been opened up for agricultural expansion, settlement and public infrastructure. Considering agricultural practices in particular, most areas consist of permanently cropped, rain-fed small fields with one additional herbaceous crop growing in sequence in the same field within one growing season and sparse tree crops. Although this is typically a humanised ecological landscape, intercropping usually done with sparse trees provides suitable niches for the tsetse [8,19]. We believe that tsetse is adapting to humanised landscapes for survival and existence. Cases of species retreating or spreading into new territories appear to be a public discovery [14,22,58]. This may explain why the model identifies some unique sections of the country as being tsetse infested although at a low scale. This outcome is important for tsetse survey and control efforts.

The strength and reliability of the assembled vector data is anchored on the properly well-suited data sourcing protocol applied. Seasonal data matching across different years was undertaken leading to the creation of wet and dry season tsetse data. This data collation approach had some weaknesses but enabled us to demonstrate the strength of the modeling tool in predicting tsetse abundance. This model can be of great value and highly reliable if accurate field based entomological data and high precision predictor variables are applied.

## Conclusion

The purpose of this research was to develop a predictive model that can reliably inform decision-makers about the tsetse abundance in Uganda, based on limited entomological survey data and a set of environmental covariates represented by vegetation and meteorological data surrogates. The study was intended to contribute to solving the data scarcity problem by providing reliable national tsetse data (maps) to guide control interventions. The need for information on the prevailing spatial patterns of tsetse fly abundance, especially in inaccessible habitats, at national and sub-national levels, with very limited resources continues to call for this kind of approach to vector mapping. It is worth observing that habitat modification by humans in the form of agricultural expansion, industrialisation, urbanisation and infrastructure development will all greatly impact and shape future tsetse fly presence and abundance in Uganda and sub-Saharan Africa in general.

There are no tsetse abundance maps for Uganda. Thus, it was not possible to compare the study results in terms of both similarities and differences with existing maps. However, under such circumstances, an attempt was made to compare the results with the available tsetse presence–absence maps by Wint [21], Albert [8] and by Ford & Katondo [12,59–61] for the purpose of identifying spatial consistency and identifying any unfamiliar or possibly new tsetse vector niches. The investigation indicated broadly comparable findings and representations, with limited species recession.

The final model revealed, with high certainty, the role of woodland vegetation, altitude, dry season temperature, dry season rainfall, dry season NDVI and game parks in shaping the tsetse abundance within Uganda. From these results, national parks will remain a source of tsetse re-infestation if no vector control is undertaken in the parks. It is also true that vector abundance will continue to be influenced by ecological factors at a finer scale, like host availability and proximity to transient or small bodies of water that were not considered in this model. These findings are expected to offer avenues for making better, spatially targeted tsetse control interventions by individuals, communities, local and central governments.

## Supporting information

S1 Protocol of data abstraction Tsetse distribution. (PDF)
