# Supplementary material for: Spatial analysis of G.f.fuscipes abundance in Uganda using Poisson and Zero-Inflated Poisson regression models
Source: PLoS Negl Trop Dis. 2021 Dec 6;15(12):e0009820. doi: 10.1371/journal.pntd.0009820 (PMC8648107; doi:10.1371/journal.pntd.0009820)
Supplement: S1 Protocol of data abstraction — (PDF) [file pntd.0009820.s001.pdf]

# Protocol of data abstraction: tsetse distribution

*Source: Cecchi et al. Parasites & Vectors (2015) 8:284   DOI 10.1186/s13071-015-0898-y*

## Tsetse occurrence

Data on tsetse occurrence are recorded in the table 'Entomo_data'. In this table, different species/subspecies detected in the same survey are recorded in separate records (one for each species/subspecies).
The table includes:

1. TSETSE_ID: A unique numeric identifier of each record in the Table. If more than one tsetse species/subspecies is detected in a survey, each tsetse species/subspecies will be assigned a different TSETSE_ID.
2. SURVEY_ID: The numeric identifier of the survey.
3. LOCATION_ID: The numeric identifier of the site where the survey was carried out (it is extracted from the corresponding field in the table 'Geo_data').
4. SOURCE_ID: The numeric identifier of the source containing the data recorded for the present survey (it is extracted from the corresponding field in the table 'Sources').
5. MONTH_ST: Starting month of the survey.
6. YEAR_ST: Starting year of the survey.
7. MONTH_EN: Ending month of the survey.
8. YEAR_EN: Ending year of the survey.
9. TRAP_TYPE: Type of trap
10. TRAP_ATTR: Odour attractant used in the traps.
11. TRAP_NO: NUMBER of traps deployed in the surveyed site.
12. TRAP_TIME: Duration of trapping (in days).
13. TRAP_NOTES: Details on the strategy of trap deployment (e.g. in which habitats were traps deployed? At what hours of the day were the traps operated? Etc…).
14. SPECIES: Name of the species or subspecies of tsetse.
15. FLIES_NO: Number of flies caught
16. FLIES_AD: Flies apparent density (flies/trap/day)
17. FLIES_AP: Flies absence/presence (Presence: Yes, Absence: No)
18. OTHER_SPECIES: presence of information on other species/subspecies in the same survey. 'Yes': other species/subspecies were detected in the same survey. 'No': no other species/subspecies was detected in the survey. 'No data': the source does not indicate whether or not other species were detected in the same survey. This field enables to extract from the database absence data at the species/subspecies-level.
19. TSETSE_INTERVENTIONS: it reports what interventions against tsetse were ongoing in the study area at the time of the survey, or in the recent past prior to the survey.
20. LONGITUDINAL: it describes whether data were extracted from a longitudinal study.
21. NOTES: Includes all important additional information as reported in the source paper.

Reference:

1. Cecchi G, Paone M, Mattioli R, Vreysen M (2015). Developing a continental atlas of the distribution and trypanosomal infection of tsetse flies (Glossina species). Parasites & vectors. 8. 284. 10.1186/s13071-015-0898-y.
